# Uncovering the Functional Link Between *SHANK3* Deletions and Deficiency in Neurodevelopment Using iPSC-Derived Human Neurons

**DOI:** 10.3389/fnana.2019.00023

**Published:** 2019-03-13

**Authors:** Guanqun Huang, Shuting Chen, Xiaoxia Chen, Jiajun Zheng, Zhuoran Xu, Abolfazl Doostparast Torshizi, Siyi Gong, Qingpei Chen, Xiaokuang Ma, Jiandong Yu, Libing Zhou, Shenfeng Qiu, Kai Wang, Lingling Shi

**Affiliations:** ^1^Guangdong-Hongkong-Macau Institute of CNS Regeneration, Ministry of Education CNS Regeneration Collaborative Joint Laboratory, Jinan University, Guangzhou, China; ^2^Department of Basic Medical Sciences, College of Medicine – Phoenix, The University of Arizona, Phoenix, AZ, United States; ^3^Department of Biomedical Informatics, Columbia University, New York, NY, United States; ^4^Children’s Hospital of Philadelphia, Philadelphia, PA, United States; ^5^Co-innovation Center of Neuroregeneration, Nantong University, Nantong, China

**Keywords:** induced pluripotent stem cells, neural stem cells, *SHANK3*, electrophysiology, RNA-Seq, autism, transcriptome

## Abstract

*SHANK3* mutations, including *de novo* deletions, have been associated with autism spectrum disorders (ASD). However, the effects of *SHANK3* loss of function on neurodevelopment remain poorly understood. Here we generated human induced pluripotent stem cells (iPSC) *in vitro*, followed by neuro-differentiation and lentivirus-mediated shRNA expression to evaluate how *SHANK3* knockdown affects the *in vitro* neurodevelopmental process at multiple time points (up to 4 weeks). We found that *SHANK3* knockdown impaired both early stage of neuronal development and mature neuronal function, as demonstrated by a reduction in neuronal soma size, growth cone area, neurite length and branch numbers. Notably, electrophysiology analyses showed defects in excitatory and inhibitory synaptic transmission. Furthermore, transcriptome analyses revealed that multiple biological pathways related to neuron projection, motility and regulation of neurogenesis were disrupted in cells with *SHANK3* knockdown. In conclusion, utilizing a human iPSC-based neural induction model, this study presented combined morphological, electrophysiological and transcription evidence that support that *SHANK3* as an intrinsic, cell autonomous factor that controls cellular function development in human neurons.

## Introduction

Autism spectrum disorders (ASD) are neurodevelopmental disorders characterized by impairments in social communication and interaction, and repetitive behaviors and restricted interests (Freitag et al., 2010; State and Levitt, 2011; Lyall et al., 2017; Rubeis et al., 2018). Multiple studies have indicated a strong connection between ASD and genetic variations of synapse-related genes and proteins, including neuroligins (*NLGNs*), postsynaptic density protein 95 (PSD-95), and SH3 and multiple ankyrin repeat domains proteins (*SHANKs*) (Xing et al., 2016; Nakanishi et al., 2017; Kathuria et al., 2018). In particular, genetic association studies have identified a significant role for *SHANK3*, which encodes a major scaffolding protein at postsynaptic densities (PSD). The *SHANK3* protein contains multiple structural domains including ankyrin repeat, Src homologous, PDZ, proline-rich, Homer binding site, sterile alpha motif (Du et al., 1998; Grabrucker et al., 2011; Monteiro and Feng, 2017; Ponna et al., 2018), through which other PSD proteins extensively interact to form the post-synaptic protein signaling complex. Genomic sequencing and exon sequencing from ASD patients have indicated a strong connection between rare mutations in *SHANK3* and ASD (Durand et al., 2007; Nemirovsky et al., 2015).

Currently, it is unclear how *SHANK3* mutations confer ASD risks by affecting the developmental trajectory of the brain, particularly the excitatory glutamatergic synapses. Several studies have utilized mouse models (Peca et al., 2011; Wang et al., 2016, 2017; Chen et al., 2017; Harony-Nicolas et al., 2017; Luo et al., 2017; Zhao et al., 2017; Amal et al., 2018; Kerrisk Campbell and Sheng, 2018; Qin et al., 2018) and neuronal cell models (Bidinosti et al., 2016; Lu et al., 2016; Bey et al., 2018; Taylor et al., 2018) to explore the role of *SHANK3* gene in synaptic function and animal behavior. The variation of *SHANK3* at different loci resulted in distinct behavioral phenotypes when modeled in mice. Most of these *SHANK3* mutant mice showed deficits in social interactions, with or without cognitive impairment, repetitive behavior, anxiety and motor deficit. Abnormal cortico-striatal circuits, disrupted excitability and inhibitory (E/I) balance, and synaptic dysfunction have been identified to be associated with the mechanism of ASD-like behavior (Bozdagi et al., 2010; Peca et al., 2011; Wang et al., 2011; Mei et al., 2016; Vicidomini et al., 2017; Bey et al., 2018). These analysis of synaptic physiology and mice behavior revealed a strong causal connection between *SHANK3* mutations and ASD-like endophenotypes.

Despite the above cited studies, analyses on animal models cannot completely simulate human genetic background on neurodevelopment. In some cases, human brain tissue sample has been collected for neural development disease study (Konopka et al., 2012; Parikshak et al., 2016), however, the sample resource is very limited. More than 10 years ago, Takahashi and Yamanaka made a remarkable breakthrough in stem cell research when they generated ES-like cells from adult somatic cells using a cocktail of transcription factors (Takahashi et al., 2007). More recently, new methods have been developed to reprogram adult somatic cells (such as fibroblasts) into iPSC. Following the discovery of iPSC, several studies have fueled enthusiasm for their use in neurological disorders, and iPSC have been validated to develop into many kinds of neural subtype cells (Chambers et al., 2009; Shi et al., 2012; Kang et al., 2017; Lin et al., 2018). Creating neuronal cultures from iPSC has received wide attention for the potential to create translatable disease-in-a-dish models. This development has made it possible to mimic human neuron development defect following disease candidate gene dysfunction. One advantage of iPSC is that the cells possess genetic background that is distinctly human; the other is that stem cells could mimic certain aspects of human neural development courses *in vitro*. So far, iPSC have been increasingly recognized as a significant *in vitro* cellular model to study the function of susceptible genes in neurological diseases and neurodevelopment diseases. iPSC have successfully used to model the cellular physiology and guide therapeutic endeavors in Rett syndrome, Alzheimer’s disease and schizophrenia by revealing the functional effects of genetic mutations with single neuron resolution (Marchetto et al., 2010; Mitne-Neto et al., 2011; Israel et al., 2012). We have also previously used iPSC models to study the functional effects of *NRXN1* deletion and *NLGN4X* deletions (Shi et al., 2013), in which we found that such synaptic genes as *NLGN4X/NRXN1* deletion directly impacts neurodevelopmental process, synaptic adhesion and neuron differentiation during the formation of neurons and their connections. *NLGNs*, which bind presynaptic *NRXNs*, are anchored in scaffold protein *SHANK3* indirectly. *SHANK3* interacts with multiple key synaptic components including glutamate receptors and their anchoring proteins, ion channels (Du et al., 1998), thus serves as a master organizer of the PSD (Naisbitt et al., 2000; Hayashi et al., 2009). *NLGNs/NRXNs/SHANK3* gene complex play an import role in synapse generation and neuron function formation.

To gain insights on the critical cellular and molecular effects of *SHANK3* in human neuron development, we generated functional neurons derived from iPSC and transduced by shRNA-based lentivirus against *SHANK3*, as well as a control shRNA. Utilizing this iPSC-based *in vitro* model, we investigated the transcriptome alteration, coupled with morphology and electrophysiological analyses to determine the impacts of *SHANK3* knockdown in the developing human neurons.

## Materials and Methods

### The Generation of iPSC-Derived Neural Development Model

Human urine epithelium-derived cells were reprogrammed into iPSC [generated by Dr. Pei’s lab (Zhou et al., 2011)]. The iPSC were cultured in Matrigel (BD Matrigel^TM^, hESC-qualified Matrix)-coated six-well plates. MTeSR^TM^ medium (Stemcell Technologies) was added to each well (2 ml per well) and replaced once a day. After reaching 95% confluence, the cells were passaged with EDTA (1:3) in Matrigel-coated 12-well plates. Once the cells density reached almost 100%, the medium was switched to neural induction medium (N_2_B_27_ + 2 inhibitors (5 μmol/L Dorsomorphin and 5 μmol/L SB431542, Selleck). Dorsomorphin and SB431542 effectively inhibit SMAD signaling pathway by blocking phosphorylation of ALK4, ALK5, ALK7 receptors and successfully improve the efficiency of neural induction. At day 8, the cells were mechanically scraped to Matrigel-coated six-well plates with neural proliferation system I (N_2_B_27_, Thermo-Fisher Scientific) medium. At day 16, the cells in 6-well cell plates were mechanically scraped to T25 flasks and were cultured with neural proliferation system II (N_2_B_27_ + 20 ng/mL bFGF + 20 ng/mL EGF, Thermo-Fisher Scientific). NPCs were expanded for three passages with Accutase (StemPro Accutase cell dissociation reagent), single cells of NPCs were directly plated at a density of 10^5^ cells per well on glass coverslips coated with glial cell feeder layer (prepared from rat astrocyte, P0–P3), and Matrigel in 24-well plates for morphology analysis. At the same time, the single cells of NPCs were directly plated at a density of 2.5 × 10ˆ^6^/well in Matrigel-coated 6-well plates for q-PCR and sequencing. The medium was switched to neuron differentiation medium (N_2_, B_27_, Thermo Fisher Scientific; 1 μM dibutyryl-cAMP, Sigma-Aldrich; 20 ng bdnf, PeproTech). The plates were kept at 37°C thereafter in a humidified incubator with 95% air and 5% CO_2_.

### Virus Transduction and Quantitative Real-Time-PCR

Single cells of NPCs were directly cultured on Matrigel-coated plates. The next day the cells were infected by shControl and shSHANK3 lentivirus in proper titers. (TRIPZ Human *SHANK3* shRNA, Dharmacon, Inc., ID: V2THS_264172, 1 × 10^8^ TU/mL). Six hours after infection, the viruses were removed and replaced with NPCs proliferation system containing doxycycline (1 μg/mL). Three days after infection, medium was switch to NPCs proliferation system containing doxycycline (1 μg/mL) and puromycin (1 μg/mL). The cells were lysed with Trizol, total RNA was extracted using an RNeasy mini kit. RNA concentration (OD_260_/OD_280_) was measured using Nanodrop 2000C Spectrophotometer. Total RNA was reverse transcribed into cDNA using the PrimeScript^TM^ RT reagent Kit with gDNA Eraser (Perfect Real Time). Real-time quantitative PCR was performed with SYBR Premix Ex Taq^TM^ II detection System and Inumin detection instrument. The primer sequences are as follows:

Human SHANK3-F, CAGGACGCGCTCAACTATG;
Human SHANK3-R, GCATAAACTCGCCGCTTGTA;
Human GAPDH-F, CATGTTCGTCATGGGTGTGAA;
Human GAPDH-R, AGTGATGGCATGGACTGTGGT.
  All data was normalized to the GAPDH mRNA level and shSHANK3 knockdown efficiency were calculated.

### Morphology Analysis

Neurons were sparsely infected with lentivirus (1 × 10^8^ TU/mL) packed with a tetracycline-controlled red fluorescent protein (RFP) expression sequence to obtain fluorescent images. Neurons plated on glass coverslips were used for morphological construction at 3–28 days after plating. Standard immunohistochemistry protocols were used. The neurons were fixed using 4% paraformaldehyde for 30 min, washed with PBS, permeabilized with 0.3% Triton X (Thermo Scientific) in PBS and blocked with 1% BSA (VETEC^TM^) in PBS. The neurons were stained by double immunofluorescence with DAPI (Thermo Scientific, D1306, 1:1000) and the following antibodies: Mouse-Oct4 (BD Pharmingen, 6765-100, 1:200), Mouse-SSEA4 (Invitrogen, 41-4000, 1:100), Mouse-SOX2 (R&D Systems, MAB2018, 1:500), Rabbit-Nestin (Millipore, ABD9, 1:1000), Mouse-TBR1(Abcam, ab31940, 1:800), Mouse-TUJ1 (Covance, Princeton, T8660, 1:1000), Tau1 (Millipore, MAB3420, 1:500), Ribbat-Map2 (2a + 2b) (Millipore, AB5622, 1:1000), Mouse-PSD95 (Thermo Fisher, MA1-045, 1:150), Rabbit-synapsin I (Millipore, AB1543, 1:1000), secondary antibodies used include: donkey anti-mouse Alexa-488 (Invitrogen, R37114, 1:1000), donkey anti-mouse Alexa-546 (Invitrogen, A10036, 1:1000). Images of neurons were visualized with 20, 40, and 60× objective (Imager Z2, Zeiss) and digitized using a Zeiss camera (Axiocam 506 mono, Zeiss). The soma area, growth cone area, neurite length and branches were analyzed and quantified with ImageJ. The complexity of neurite arborization was analyzed with the ImageJ/FIJI. Statistic results were analyzed by two-way ANOVA with GraphPad Prism 5.01. Growth cone was categorized into three types (blunt ended, filopodial and lamellipodial) as described by Khazaei et al. (2014).

### Electrophysiological Recording

The RFP-expressing iPSC-derived neurons were perfused with artificial spinal cerebral fluid (pH 7.4, in mM: 126 NaCl, 2.5 KCl, 2 CaCl_2_, 2 MgCl_2_, 26 NaHCO_3_, 1.25 NaH_2_PO_4_ and 10 D-glucose) bubbled with 95% O_2_ and 5% CO_2_ at RT (25 ± 1°C). The neurons were visualized with a 40× water objective on an IR-DIC microscope (Nikon Eclipse FN-1 microscope) and recorded using an amplifier (MultiClamp 700B, Molecular Devices). Borosilicate microelectrodes with a resistance of 4–8 MΩ were pulled using a pipette puller (Narishige PC10) and the glass pipette was filled with filtered intracellular recording solution (pH 7.3, 290–310 mOsm, in mM: 126 *K*-gluconate, 4 KCl, 0.3 Na_2_-ATP, 4 Mg-GTP, 10 phosphocreatine, 10 HEPES). Recordings were filtered at 3 kHz (low pass) and digitized at 20 kHz (DigiData 1550A, Molecular Devices), and statistical analysis of electrophysiology data was collected and analyzed with pClamp10 (Molecular Devices). The input resistance of the cells (*R*_in_) was recorded as the slope of linear fits of current-voltage plots responded to 1 s current injection steps (−10, 2, or 3 pA steps) in current-clamp mode. Cells were held at resting membrane potential (RMP), not corrected for a liquid junction potential. Spontaneous excitatory (EPSCs) and inhibitory (IPSCs) postsynaptic currents were recorded at a −70 and 0 mV, respectively, for 3 min in voltage-clamp mode. sEPSCs and sIPSCs were verified by complete blockade of kynurenic acid (KyA, 3 mM, Sigma-Aldrich) or SR95531 (10 μM, Tocris). The sodium currents (*I*_Na_) were recorded at −70 mV in voltage-clamp mode and elicited by 200 ms voltage steps from −20 to 50 mV at 5 mV increments.

### Illumina Transcriptome Library Preparation and Sequencing

The total RNA was subject to RNA-Seq analysis. RNA concentration was measured using Nanodrop 2000C Spectrophotometer and Qubit 3.0 (Invitrogen). RNA integrity number and the library length were detected by BioAnalyzer 2100 (Agilent). Library construction was performed with Truseq RNA Access library Pre Kit (Illumina, RS-301-2001). The library was sequenced on a HiSeq X platform (Illumina).

### Western Blot Analysis

Cultured cells were washed twice with PBS (1 mL/well), then 200 μl cell lysis buffer (RIPA buffer (Thermo scientific) supplemented with protease inhibitor (Calbiochem, 50:1) were added to each well. Cells were collected with a cell scraper (NEST Biotechnology). Cell suspension was collected and allowed to stand on ice for 30 min, and then centrifuged at 4°C, 14,000 rpm for 15 min. After centrifugation, supernatant was transferred to a new tube and protein concentration was quantified using Pierce^TM^ BCA Protein Assay Kit (Thermo Fisher Scientific). Samples were mixed with 10× loading buffer (Takara) and boiled for 10 min 20 μg proteins were loaded each lane and separated by SDS-PAGE gel. The proteins were then transferred to PVDF membranes (Millipore), incubated with an antibody against *SHANK3* protein (Santa Cruz Biotechnology), diluted in 5% BSA overnight at 4°C. The PVDF membranes were then washed three times, and further processed with HRP-conjugated secondary antibodies for 2 h at room temperature. Protein signals were developed using ChemiDoc Touch Imaging System. The optical density of immunoreactive bands was quantified by ImageJ.

### Bioinformatics Analysis of RNA-Seq Data

All the sequencing data were obtained as FASTQ files for 16 samples with four different measurement times at days 0, 7, 9, and 28. To improve the quality of alignment, we used FastQC (Andrews, 2010) to investigate the quality of reads in the dataset and Cutadapt (Martin, 2011) for removing adapter sequences. The reads were then mapped to the reference human genome (GRCh38) and gene count data were produced using STAR (Dobin et al., 2013). Then, we proceeded with downstream analysis after assuring that the rates of uniquely mapped reads for all samples were higher than 80%.

Generalized linear model was used to identify differentially expressed genes between *SHANK3* knockdown group and the control group over time using DESeq2 (Love et al., 2014). MA-plot (Supplementary Figure S6) and dispersion estimate plot (Supplementary Figure S7) were generated for quality control before further analysis. After performing regularized-logarithm transformation on the raw counts data using *rlog* function in DESeq2, we plotted a sample hierarchical clustering heatmap using Euclidian distance to assess the similarity among samples. The limma R package (Ritchie et al., 2015) was used for correcting batch effects among the samples produced from two different sequencing experiments. Benjamini and Hochberg correction methods (Benjamini and Hochberg, 1995) for multiple-testing adjustment were adopted in moderated *t*-test to identify differentially expressed genes. The genes with adjusted *p*-value less than 0.05 were selected as differentially expressed genes, which represent the ones at one or more time points with a gene-specific difference accounting for the difference at time 0. Then Kyoto Encyclopedia of Genes and Genomes (KEGG) pathways and Gene Ontology (GO) enrichment analysis were performed for function annotation on WebGestalt (Wang et al., 2013), and FDR < 0.01 was used as the threshold to examine significant results.

## Results

### Generation of Neural Development Model *in vitro*

To study the effect of *SHANK3* loss of function on neurodevelopment, we generated iPSC-derived neural development model *in vitro* with two-inhibitor culture system (Dorsomorphin and SB4315242) and collected neural cells for knockdown efficiency or morphology analysis at multiple time points (days 3, 7, 9, 14, 21, and 28). The iPSC were induced into neural progenitor cells (NPC), which were cultured in floating, and then differentiated into neurons on coverslips. The 1st day of induction on coverslip was defined as day 0 (D0).

**FIGURE 1:**
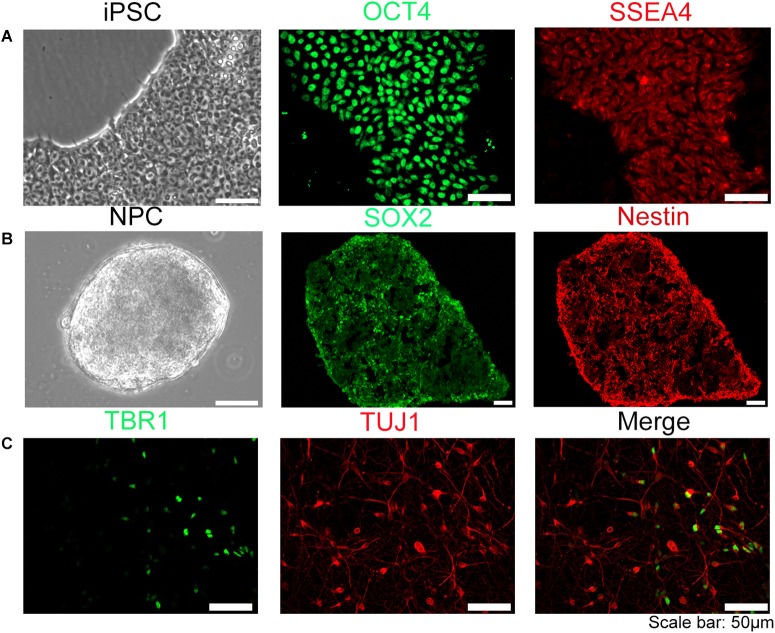
Establishment of human iPSC model. The iPSC were induced into neural progenitor cells (NPC) and further differentiated into neurons. **(A)** iPSC used in our experiment were OCT4, SSEA4 positive stain. **(B)** NPC induced form iPSC were SOX2, Nestin positive. **(C)** Neurons differentiated from NPC were TUJ-1 or TBR1 positive, suggesting that NPC can be differentiated into multiple categories of neural cells. Scale bar: 50 μm.

The human iPSC used in this experiment were stained positive with specific iPSC marker proteins (OCT4 and SSEA4) (Figure 1A) and specific neural stem cells marker proteins (SOX2, nestin) (Figure 1B), indicating that the cells have the potential of differentiating into neurons. NPC were directly plated on glass coverslips coated with rat glial cell feeder layer and Matrigel in 24-well plates for sample collection in different time points. The neuron-specific marker protein TUJ-1, Tau-1, Map2, deep layer cortical neuron marker protein TBR1, and subtype neuron marker protein VGLUT1, GABA, TH were detected by immunohistochemistry (Figure 1C and Supplementary Figure S1). The results showed that neurons derived from iPSC could be differentiated into multiple CNS neuron types, reflecting a highly induction efficiency (estimated > 90%) using our method. We have further quantified that in this *in vitro* induced neural model, the proportion of glutamatergic neurons is about 10.7%, dopaminergic neurons is about 17.3%, GABAergic neurons is approximately 54.9%.

*SHANK3* expression pattern data from Allan Brain Institute pointed to abundant expression in the developing brain at very early development stage^1^ (Supplementary Figure S2A), that is consistent with our Western blot result of *SHANK3* (Supplementary Figure S2B). We also found that expression of *SHANK3* emerged in early neuronal development stages (P0-7), and continue to increase into young adulthood (P42). This increased levels of *SHANK3* protein may reflect the time course of cortical synapse maturation (Supplementary Figures S2A,B).

### Morphological Effects of *SHANK3* Knockdown on Neurodevelopment

**FIGURE 2:**
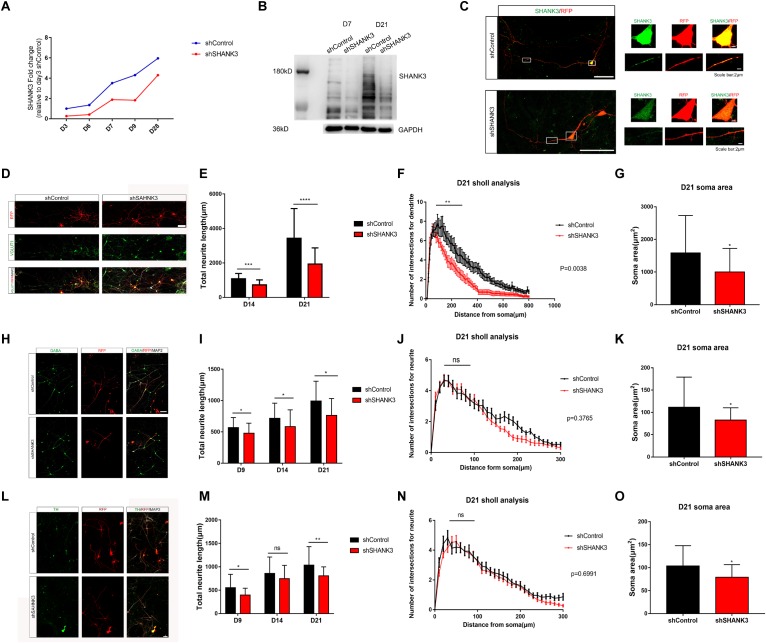
*SHANK3* knockdown profoundly affects neurite length, complexity of neurite arborization and soma area. **(A–C)** The efficiency of *SHANK3* knockdown in iPSC-derived neurons was verified by qRT-PCR (D3, D7, and D28), Western blot (D7 and D21), and immunofluorescence staining (D28). **(D,H,L)** Immunofluorescence staining of VGLUT1, GABA, TH, and MAP2 confirms generation of multiple types of differentiated neurons in our culture system. Scare bar: 50 μm. **(E,I,M)** Comparison of neurite length in neurons infected with shShank3 and shControl viruses. Data are shown as mean ± SEM. ^∗^*p* < 0.05, ^∗∗^*p* < 0.01, ^∗∗∗^*p* < 0.001, and ^∗∗∗∗^*p* < 0.0001. (D9: GABA: *n* = 33 control and 35 *SHANK3* knockdown cells, *p* = 0.0181; TH: *n* = 33 control and 33 *SHANK3* knockdown cells, *p* = 0.0159); D14: VGLUT1: *n* = 22 control and 22 *SHANK3* knockdown cells, (*p* = 0.0003), GABA: *n* = 33 control and 34 *SHANK3* knockdown cells (*p* = 0.0219), TH: *n* = 34 control and 37 *SHANK3* knockdown cells (*p* = 0.2482); D21: VGLUT1: *n* = 22 control and 24 *SHANK3* knockdown cells (*p* < 0.0001), GABA: *n* = 30 control and 29 *SHANK3* knockdown cells (*p* = 0.0037), TH: *n* = 30 control and 30 *SHANK3* knockdown cells, (*p* = 0.0035). **(F,J,N)** Analysis of the effect of *SHANK3* gene knockdown on the complexity of neurite arborization. Statistical significance was evaluated by two-way ANOVA, *p* = 0.0038 (D21), *p* = 0.3765 (D21), *p* = 0.6991 (D21), data are shown as mean ± SEM. ^∗^*p* < 0.05, ^∗∗^*p* < 0.01, ^∗∗∗^*p* < 0.001. **(G,K,O)** Statistic analysis of the effect of *SHANK3* knockdown on neuronal soma area. Statistical significance was evaluated by two-way ANOVA, data are shown as mean ± SEM. ^∗^*p* < 0.05, ^∗∗^*p* < 0.01, ^∗∗∗^*p* < 0.001. (D21: VGLUT1: *n* = 22 control and 24 *SHANK3* knockdown cells, GABA: *n* = 30 control and 29 *SHANK3* knockdown cells, TH: *n* = 30 control and 30 *SHANK3* knockdown cells).

We have generated two sets of neural induced model from two separate iPSC lines in this whole project, and we used one clone of each line to collect data for morphology analysis (Supplementary Figure S3). To knockdown *SHANK3* expression in NPC, we infected NPC with shRNA-based lentivirus against *SHANK3* or with a control lentivirus, here after referred as shSHANK3 and shControl, respectively. RFP signals were observed in 1 week post infection. Knockdown efficiency was verified at multiple levels, including qRT-PCR (Figure 2A), Western blot analysis (Figure 2B) and immunocytochemistry staining of cultured neurons (Figure 2C). These data suggest lentiviral-mediated *SHANK3* knockdown was highly effective.

We next investigated the impact of *SHANK3* knockdown on neurite length. Neuron morphology was traced and reconstructed by ImageJ. Sholl analysis was used to quantify neurite complexity. We further classified neurons into three groups in our *in vitro* model, excitatory neurons (VGLUT1^+^), inhibitory neurons (GABA^+^), and putative dopamine neurons (TH^+^) (Supplementary Figure S1). To distinguish axons and dendrites, we used MAP2 and Tau1 antibody to co-stain dendrites (MAP2^+^) and axons (Tau1^+^) (Supplementary Figure S1). Neuronal morphology was constructed base on RFP expression. We examined the effects of *SHANK3* knockdown on neurite development (e.g., length and the number of branches) at different time points. We observed that, the total dendrites length of VGLUT1 positive neuron decreased significantly with *SHANK3* knockdown on D14 (*p* = 0.0003), D21 (*p* < 0.0001) (Figure 2D,E); GABA positive neuron dendritic length decreased significantly on D9 (*p* = 0.0181), D14 (*p* = 0.0219), D21 (*p* = 0.0037) (Figure 2H,I); while TH positive neuron dendritic length decreased significantly on D9 (*p* = 0.0159), D14 (*p* = 0.2482), D21 (*p* = 0.0035) (Figure 2L,M). These results indicate that *SHANK3* gene is required for the development of neurite length in iPSC derived human neurons.

To further assess of neurite complexity, Sholl analysis was used to quantify neurite branches number. We observed that *SHANK3* knockdown had significant effect on neurite complexity in excitatory neurons at D21 (Figure 2F), especially on the neurite branches number close to the cell body area within 100 microns, while *SHANK3* deficit had few effect on neurite complexity of inhibitory neurons and dopamine neurons (Figure 2J,N). These results indicate that *SHANK3* gene is required for the development of excitatory neuronal branches in iPSC-derived human neurons.

We next investigated the morphological differences following *SHANK3* knockdown in iPSC-derived human neurons, we analyzed soma and growth cone development in shControl and shShank3 groups. We observed that soma area of VGLUT1 positive neuron significantly decreased with *SHANK3* knockdown at D21 (*p* = 0.0097), the soma area of GABA positive neurons decreased at D9 (*p* = 0.0254), D21 (*p* = 0.0108), the soma area of TH positive neurons decreased at D9 (*p* = 0.0294), D14 (*p* = 0.0002) (Figure 2G,K,O). We have identified TH and GABA positive neuronal soma size in D9, as VGLUT1 antibody specifically stained excitatory neuron at least in D14 in our study. The results indicate that *SHANK3* gene knockdown affects the development of neuronal soma in early neuronal development of D9, at least in GABA and TH positive neurons. In addition, *SHANK3* knockdown has a significant effect on VGLUT1 positive neuron in D21.

**FIGURE 3:**
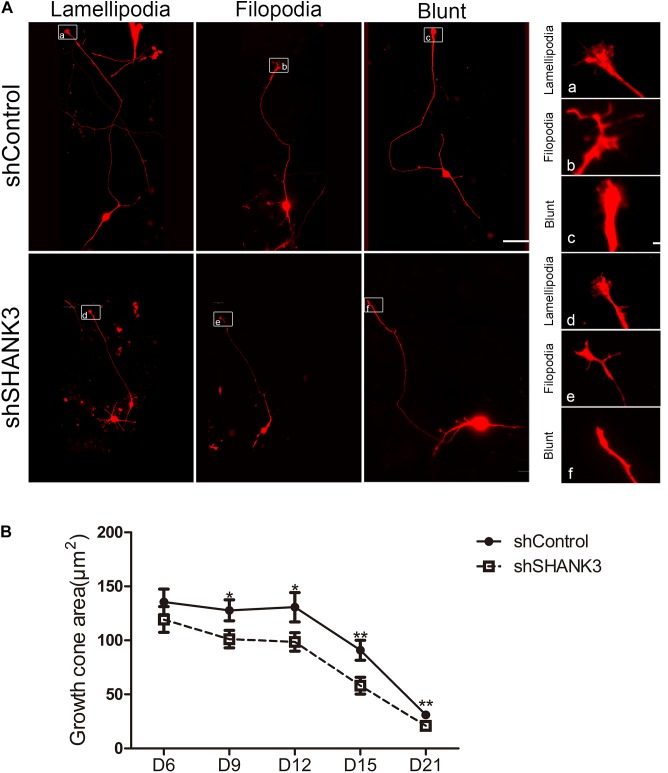
The effects of *SHANK3* knockdown on neurons growth cone area. **(A)** Representative images of the growth cones of shControl and shSHANK3 transduced neurons. Scare bar: schematic diagram 50 μm; enlarged area 5 μm. **(B)** Analysis of the effect of *SHANK3* knockdown on the growth cone area of neurons. Statistical significance was evaluated by two-way ANOVA, date are shown as mean ± SEM. ^∗^*p* < 0.05, ^∗∗^*p* < 0.01. (D6: *n* = 57; D9: *n* = 67 control and 62 *SHANK3* knockdown cells; D12: *n* = 64 control and 63 *SHANK3* knockdown cells; D15: *n* = 65 control and 61 *SHANK3* knockdown cells; D21: *n* = 67 control and 64 *SHANK3* knockdown cells; D28: *n* = 65).

Next, we examined whether *SHANK3* is required for growth cone formation. We found that the area of the growth cone significantly decreased with *SHANK3* knockdown at all-time points (days 9, 12, 15, and 21) (Figure 3). These results indicate that *SHANK3* knockdown affects the development of neuronal growth cone area.

### *SHANK3* Knockdown Affects the Electrophysiological Properties of Developing Human Neurons

**FIGURE 4:**
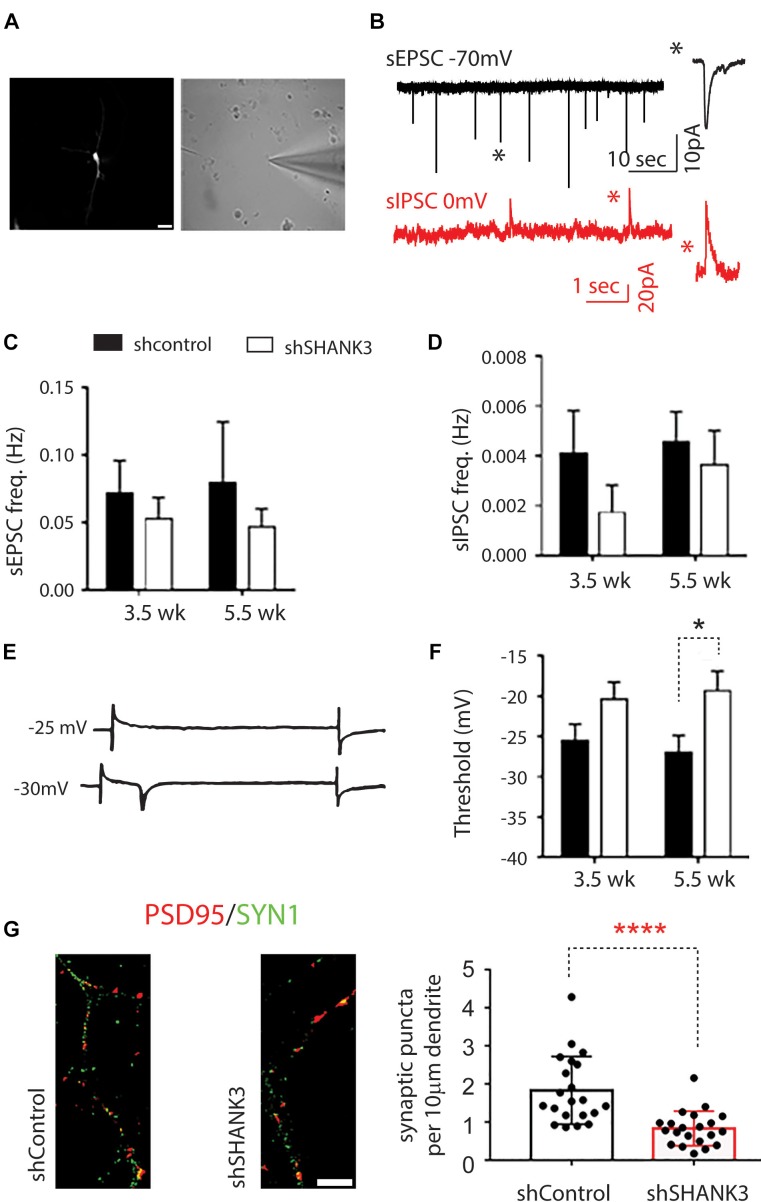
The effects of *SHANK3* gene knockdown on neurons function. **(A)** RFP-expressing cell visualized under differential interference contrast microscopy. **(B)** Sample recordings of sEPSC (top panel) and sIPSC (bottom panel). **(C)** Averaged frequency of spontaneous EPSCs at 3.5 and 5.5 weeks in culture (3.5 weeks: *n* = 17 control and 12 *SHANK3* knockdown cells; 5.5 weeks: *n* = 11 control and 15 *SHANK3* knockdown cells). **(D)** Averaged frequency of spontaneous IPSCs (3.5 weeks: *n* = 10 control and 7 *SHANK3* knockdown cells; 5.5 weeks: *n* = 15 control and 13 *SHANK3* knockdown cells). **(E)** Illustration of sodium currents elicited by voltage step commands. **(F)** The threshold of sodium currents (3.5 weeks: *n* = 19 control and 12 *SHANK3* knockdown cells; 5.5 weeks: *n* = 15 control and 15 *SHANK3* knockdown cells). ^∗^*p* < 0.05. **(G)** Synapse number changes following *SHANK3* knockdown: PSD95 (GFP) and Synapsin I (RFP) co-staining showed the synaptic puncta was reduced after *SHANK3* knockdown (*n* = 21, ^∗∗∗∗^*p* < 0.0001).

To investigate functional outcomes of *SHANK3* knockdown, we conducted whole cell patch clamp recording on RFP-expressing neurons at 3.5 and 5.5 weeks (Figure 4). To determine if excitatory synaptic transmission was altered in neurons with *SHANK3* knockdown, were recorded spontaneous excitatory post-synaptic currents (sEPSCs). We found that the frequency of sEPSC from *SHANK3* knockdown neurons was reduced relative to control neurons at 3.5 and 5.5 weeks (Figure 4C). In addition, spontaneous inhibitory post-synaptic currents (IPSCs) were recorded to determine the effect of *SHANK3* down-regulation in inhibitory synaptic transmission. We found that the frequency of sIPSC recorded from *SHANK3* knockdown neurons was reduced relative to control neurons at 3.5 and 5.5 weeks (Figure 4D). Taken together, these results suggest that both excitatory synaptic transmissions and inhibitory synaptic transmission are impaired in neurons with *SHANK3* knockdown.

Next, we investigated sodium currents threshold (*I*_Na_). The results showed that neurons with *SHANK3* knockdown exhibited an increase in the threshold of sodium currents at 3.5 weeks and increased significantly (*p* = 0.2245) relative to control neurons at 5.5 weeks (Figure 4F), suggesting that down-regulation of *SHANK3* may extensively alter receptors for neurotransmission and voltage gated ion channels.

### Effects of *SHANK3* Knockdown on Transcriptome Regulation

**FIGURE 5:**
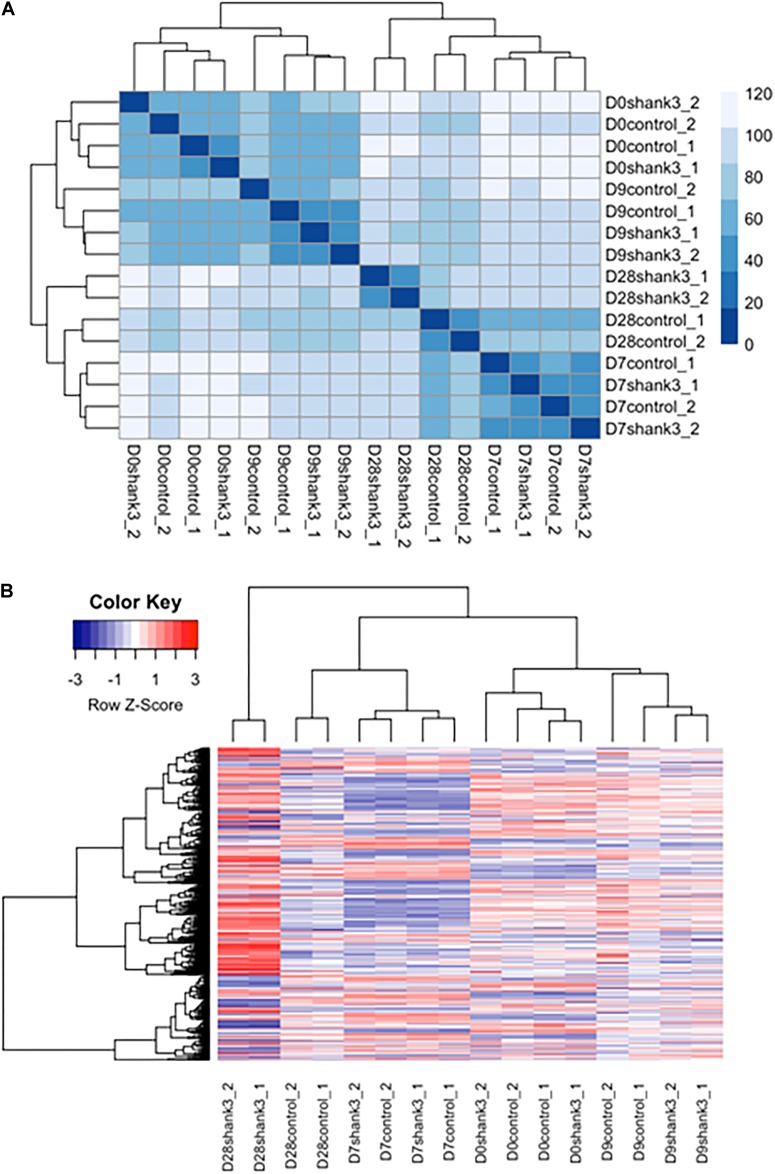
Heatmap of differentially expressed genes associated with *SHANK3* loss of function. **(A)** Euclidian distances were used for constructing sample distance matrix. After eliminating batch effect that would separate the samples from two different experiments apart, samples measured at the same time points were clustered together over influence of gene knockdown. **(B)** Heatmap for 1576 differentially expressed genes (*p*-adj<0.05) over time. It can be seen obviously that the expression difference mainly occurred at day 28.

We first examined the overall similarity between samples by using hierarchical clustering. It can be seen from the heatmap (Figure 5A and Supplementary Figure S8) that samples from the same measurement time points were clustered together regardless of the *SHANK3* knockdown status. These results indicate that time exerted more important influence in determining gene expression variation comparing with *SHANK3* knockdown.

Next, differentially expressed genes were estimated between *SHANK3* knockdown and control groups across four measurement time points (Supplementary Table S1). The main question is whether *SHANK3* knockdown can alter gene expression over time and which genes would show this gene-specific differences. Considering the correlation of the response variable among different measurement time points and batch effects from two different experiments, we designed a generalized linear model with independent variables of batch, time, *SHANK3* knockdown and an interaction term with *SHANK3* and time. By testing the interaction term with likelihood ratio test, we identified 1576 differentially expressed genes (Supplementary Figure S5) over time with adjusted *p*-value less than 0.05 (*p*-adj < 0.05). Among those overall differentially expressed genes, 1078 genes showed significant difference at day 28, which can be seen obviously in the heatmap of differentially expressed genes (Figure 5B). The down regulated expression level of *SHANK3* gene and 9 genes with smallest *p*-adj values are shown in Supplementary Figure S5.

Next, we examined whether these top differentially expressed genes shared common pathways or functional categories. We performed over representation enrichment analysis for pathways and gene ontology (Supplementary Figure S4). The results showed that *SHANK3* down-regulation led to hippo signaling pathway (Hsa04390, FDR = 1.33e-05) and focal adhesion (Hsa04510, FDR = 1.84e-08) abnormalities. In addition, terms in GO analysis including neuron projection development (GO: 0031175, FDR = 0e+00, BP), regulation of neurogenesis (GO: 0050767, FDR = 0e+00, BP), central nervous system development (GO: 0007417, FDR = 0e+00, BP), focal adhesion (GO: 0005925, FDR = 1.2e-09, CC) and calcium ion binding (GO: 0005509, FDR = 4.7e-11, MF) were significantly enriched (Table 1). These transcriptome data, in combination with the morphological and functional alternations, suggest *SHANK3* has a previously uncircumscribed role in developing neurons in addition to its function as a postsynaptic scaffold protein in mature synapse.

**Table 1:** Affected signaling pathways and gene ontology categories.

GO	Name	FDR	Observed counts	Expected counts	Enrichment ratio	*P*-value
Has:04390	Hippo signaling pathway	1.33e−05	32	11.84	2.7	1.32e−07
Has:04510	Focal adhesion	1.84e−08	45	16.16	2.78	1.21e−10
GO:0031175	Neuron projection development	0e+00	132	62.24	2.12	0e+00
GO: 0050767	Regulation of neurogenesis	0e+00	119	51.79	2.3	0e+00
GO:0007417	Central nervous system development	0e+00	143	68.37	2.09	0e+00
GO: 0005925	Focal adhesion	1.2e−09	60	23.7	2.53	2.34e−11
GO:0005509	Calcium ion binding	4.7e−11	106	49.32	2.15	2.6e−14

## Discussion

In this study, we investigated the impact of *SHANK3* loss of function in neurodevelopment by combining morphology, electrophysiology and transcriptomics analyses in neurons derived from iPSC. *SHANK3* is a PSD components of glutamatergic synapses that plays a key role in excitatory synaptic transmission in adult brain (Rubeis et al., 2018). There is strong indication that *SHANK3* gene mutations or variations are associated with increased autism risks, *SHANK3* point mutations, truncations, and disruption by chromosome translocation have been all reported in ASD cases (Durand et al., 2007; Gauthier et al., 2009; Sykes et al., 2009; Peca et al., 2011; Boccuto et al., 2013). Consistently, mice mutant for *SHANK3* display multiple neurological deficits including compulsive and repetitive behaviors, that are associated with deficits in corticostriatal circuits (Peca et al., 2011; Xiaoming et al., 2011; Bariselli et al., 2016; Sebastiano and Camilla, 2016; Zhou et al., 2016; Bariselli and Bellone, 2017; Jin et al., 2018). However, the molecular targets of *SHANK3* that are causally linked to the ASD-like behavioral deficits, and how disruption of *SHANK3* derails the normal neurodevelopmental trajectory in human brain remain largely unclear. In this study, we took advantage of an iPSC neuron induction model, and used shRNA to induce *SHANK3* loss of function to investigate the neurodevelopmental role of *SHANK3* protein in human neurons. Consistent with previous reports (Durand et al., 2012), we found that synapse numbers were reduced, and synaptic transmissions are impaired in *SHANK3* knockdown neurons.

It’s worth noting that *SHANK3* protein expressed in early developing stage from NPC to mature neuron based on our qRT-PCR and Western blot results. This is consistent with the curated data on the Allen Brain Atlas, which show that *SHANK3* mRNA is abundant (Supplementary Figure S2). This early *SHANK3* expression suggests a functional role in developing neuronal morphology and emergence of function, in addition to being a postsynaptic scaffold protein at the mature glutamatergic synapses at later developmental stages. We also found that *SHANK3* deficiency affects the morphology at early development stages, before the synaptic transmission are established and mature. Our results show that *SHANK3* knockdown reduces dendritic arborization in three major types of differentiated neurons (glutamatergic/GABAergic/dopamine). We also found that the soma size and growth cone area of glutamatergic/GABAergic/dopamine neuron were reduced as a result. We further confirmed *SHANK3* protein did localize in dendrite and soma by immunofluorescence staining experiments. A few previous studies have reported that *SHANK3* localization in neuron soma, dendrite, growth cone (Du et al., 1998; Boeckers et al., 2002; Durand et al., 2012; Halbedl et al., 2016) and the dendritic deficits (Durand et al., 2012) following *SHANK3* deficiency in human neurons. *SHANK3* protein is found to be located at the tip of actin filaments and promotes growth cone motility in developing neuron by enhancing actin polymerization (Durand et al., 2012; Halbedl et al., 2016). A *de novo SHANK3* mutation in the ankyrin domain (Q312R) is associated with growth cone formation and motility in animal models (Durand et al., 2012; Kathuria et al., 2018). Kathuria et al. (2018) uncovered the *SHANK3*’s critical role in neuronal morphogenesis and the early defects which are associated with ASD-associated mutations. Our data are consistent with previous studies. Morphology analyses indicates strong correlation between *SHANK3* knockdown and dendritic branching abnormalities; neuron soma area, neurite number and length, complexity of neurite arborization and growth cone area are all significantly affected by *SHANK3* knockdown.

Another important finding from this study is that *SHANK3* knockdown changes the transcriptome landscape across the time course of neural development. We identified genes with temporal changes of expression patterns across four time points between control shRNA and the shSHANK3 groups, and performed pathway enrichment analysis. We showed that even in early stage, the development-associated pathways can be affected by *SHANK3* knockdown, which is consistent with the observed abnormality of neuronal morphology following *SHANK3* knockdown in early stage of neurodevelopment. The PI3K associated pathway has been enriched in transcriptome analysis in our study, which also support the hypothesis that *SHANKs* mutation serves to cross-link further disease related signaling cascades (mTOR/PI3K). Altogether, these results indicate that *SHANK3* has a previously unappreciated role in early neuronal development, in addition to its well established functional role as a postsynaptic scaffold protein in the mature synapse.

Our electrophysiology results are generally consistent with the morphological findings. We found that both excitatory synaptic transmissions and inhibitory synaptic transmission are impaired in *SHANK3* knockdown neurons. However, the underlying mechanism of the synaptic transmission impairment in *SHANK3* knockdown neurons should be investigated in future studies. Taken together, our study show *SHANK3* loss of function profoundly derails the developmental trajectory of human neurons. Many functional domains of neural development are impaired, including dysplasia of neuronal soma, stunted neurite and growth cone, and altered of transcriptome. These changes suggest *SHANK3* loss of function as an intrinsic, cell autonomous factor that impairs cellular development in human neurons both in early and in mature stages, which may account for the brain pathological changes in neurodevelopmental diseases such as ASD.

## Author Contributions

LS designed the study and contributed to transcriptome data analysis, wrote and approved the final version of the manuscript. GH, SC, XC, QC, ZX, SG, and XM conducted cell culture experiments, neural morphology, and other data analysis. GH and SC co-wrote the final version of the manuscript. JZ and JY generated the electrophysiological data. KW and AT contributed to performing transcriptome data analysis. LZ and SQ contributed to the experimental design, data analyses, and preparation of the manuscript.

## Conflict of Interest Statement

The authors declare that the research was conducted in the absence of any commercial or financial relationships that could be construed as a potential conflict of interest.

## Footnotes

1 http://developingmouse.brain-map.org/

## Supplementary Material

FIGURE S1 Expression of MAP2 (GFP), Tau1 (RFP), VGLUT1 (GFP), GABA (GFP), and TH (GFP) in D28 neurons in our *in vitro* model, further supporting successful differentiation into various mature neuron types.

FIGURE S2 Expression of *SHANK3* mRNA and protein at very early developmental stages in mouse brain. **(A)** *SHANK3* mRNA is abundantly expressed in the developing brain at early development stage (source: Allen Brain Atlas). **(B)** *SHANK3* protein can be detected by Western blot in developing mouse cortex tissues as early as P0, but increase as the brain matures.

FIGURE S3 Two sets of neural induced model from two separate iPSC lines have been generated in this whole project, and we used one clone of each line to collect data for morphology analysis. Both of these two clone showed significant decrease on dendritic length and soma size after *SHANK3* knockdown. Comparison of neurite length in another iPSC line neurons infected with shShank3 and shControl viruses. Morphology reconstructed with ImageJ, statistical significance was evaluated by *T*-test, data are shown as mean ± SEM. ^∗^*p* < 0.05. **(A)** D9: GABA: *n* = 35 control and 33 *SHANK3* knockdown cells, (*p* = 0.0037); **(B)** D9: TH: *n* = 30 control and 31 *SHANK3* knockdown cells, (*p* = 0.0340); **(C)** Detailed Vector Map of the pTRIPZ lentiviral vector.

FIGURE S4 Bar charts of gene ontology function categories.

FIGURE S5 Effects of *SHANK3* knockdown on gene expression levels. Expression level were transformed into log10 scale. Green lines represent *SHANK3* knockdown group and red lines represent control group. **(A)** Expression level of *SHANK3* gene over time between knockdown and control group. **(B)** Expression level of top 9 genes with smallest *p*-adj values.

FIGURE S6 RNA sequence data MA plot. Each dot represents a gene. *X*-axis is the average expression over all samples. *Y*-axis is the log2 fold change between *SHANK3* knockdown and control group. Genes with FDR<0.01 are shown in red. This plot demonstrates that only genes with a large average normalized count contain more information to yield a significant call.

FIGURE S7 Dispersion estimation plot for raw counts data. The dispersion fit is an exponentially decaying curve where dispersion decreased as the counts increased of all genes. Each black point represents the dispersion estimates for each gene across all the eight samples. The red line is fitted trend line, which shows the dispersions’ dependence on the mean. Blue points are the final estimates from black points shrunk to the red fitted line. The blue circles are genes which are labeled as dispersion outliers and are not shrunk toward the fitted trend line. *X*-axis is the average expression over all eight samples. *Y*-axis is the dispersion value. The dispersion fit for all genes is an exponentially decaying curve where dispersion decreased with the mean of normalized counts.

FIGURE S8 The list of top 150 differentially expressed (DE) at each time point between the knockdown and control samples.

TABLE S1 Differential expression between SHANK3 knockdown and control groups.
