# Supplementary figures and images for: Uncovering the Functional Link Between SHANK3 Deletions and Deficiency in Neurodevelopment Using iPSC-Derived Human Neurons
Source: Front Neuroanat. 2019 Mar 13;13:23. doi: 10.3389/fnana.2019.00023 (PMC6424902; doi:10.3389/fnana.2019.00023)

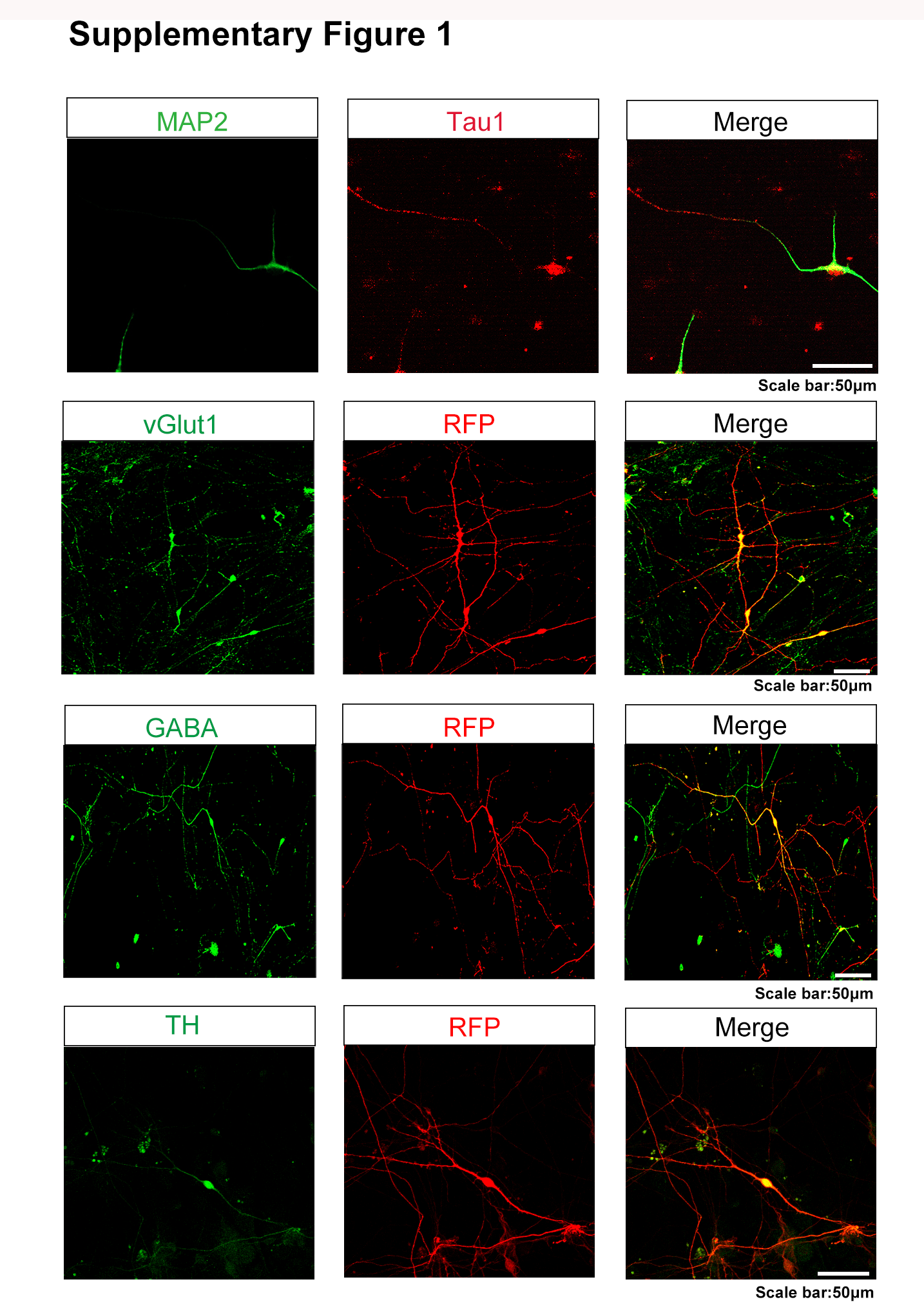

Supplement: FIGURE S1 — Expression of MAP2 (GFP), Tau1 (RFP), VGLUT1 (GFP), GABA (GFP), and TH (GFP) in D28 neurons in our in vitro model, further supporting successful differentiation into various mature neuron types. [file Image_1.TIF]

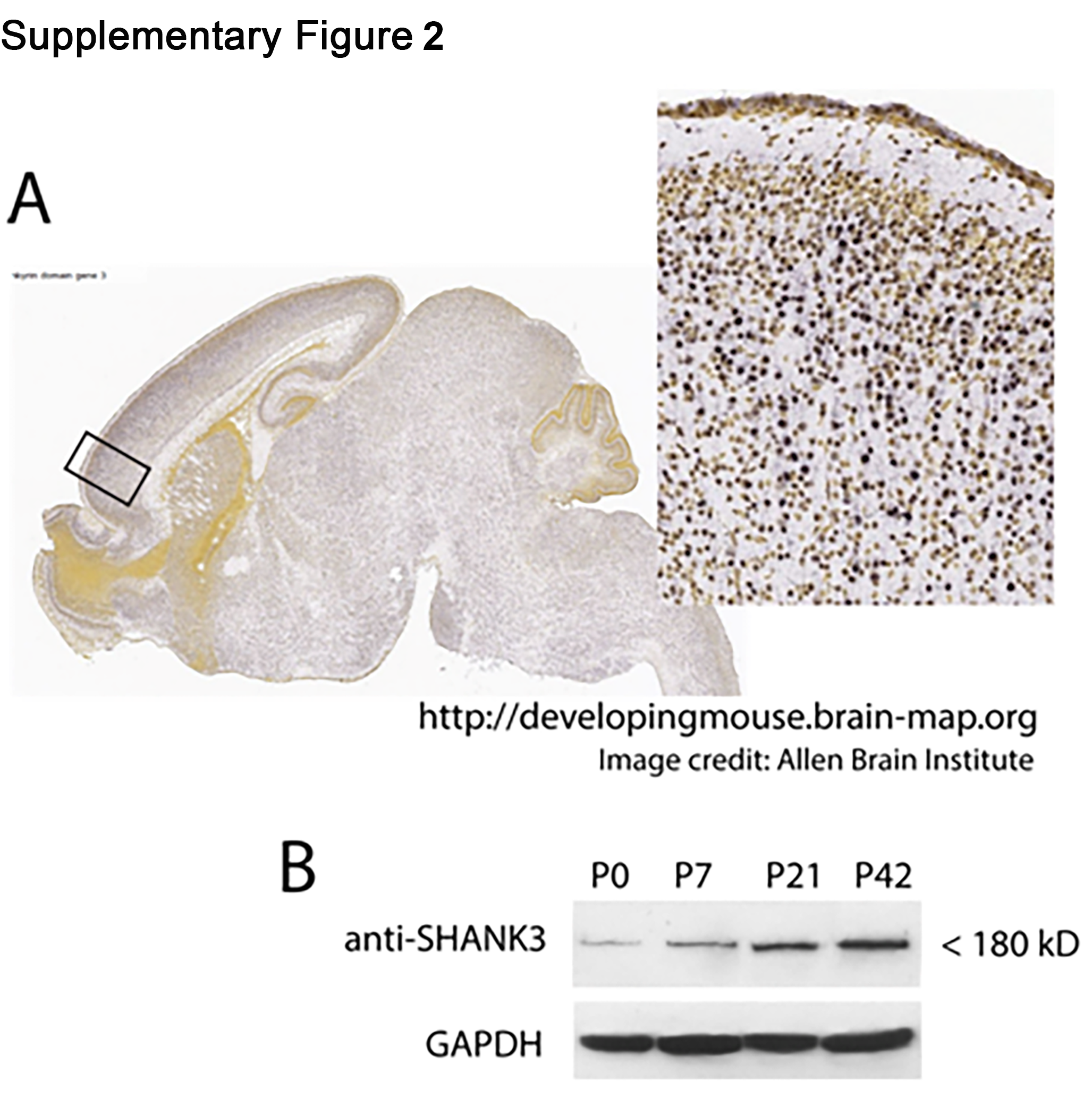

Supplement: FIGURE S2 — Expression of SHANK3 mRNA and protein at very early developmental stages in mouse brain. (A) SHANK3 mRNA is abundantly expressed in the developing brain at early development stage (source: Allen Brain Atlas). (B) SHANK3 protein can be detected by Western blot in developing mouse cortex tissues as early as P0, but increase as the brain matures. [file Image_2.TIF]

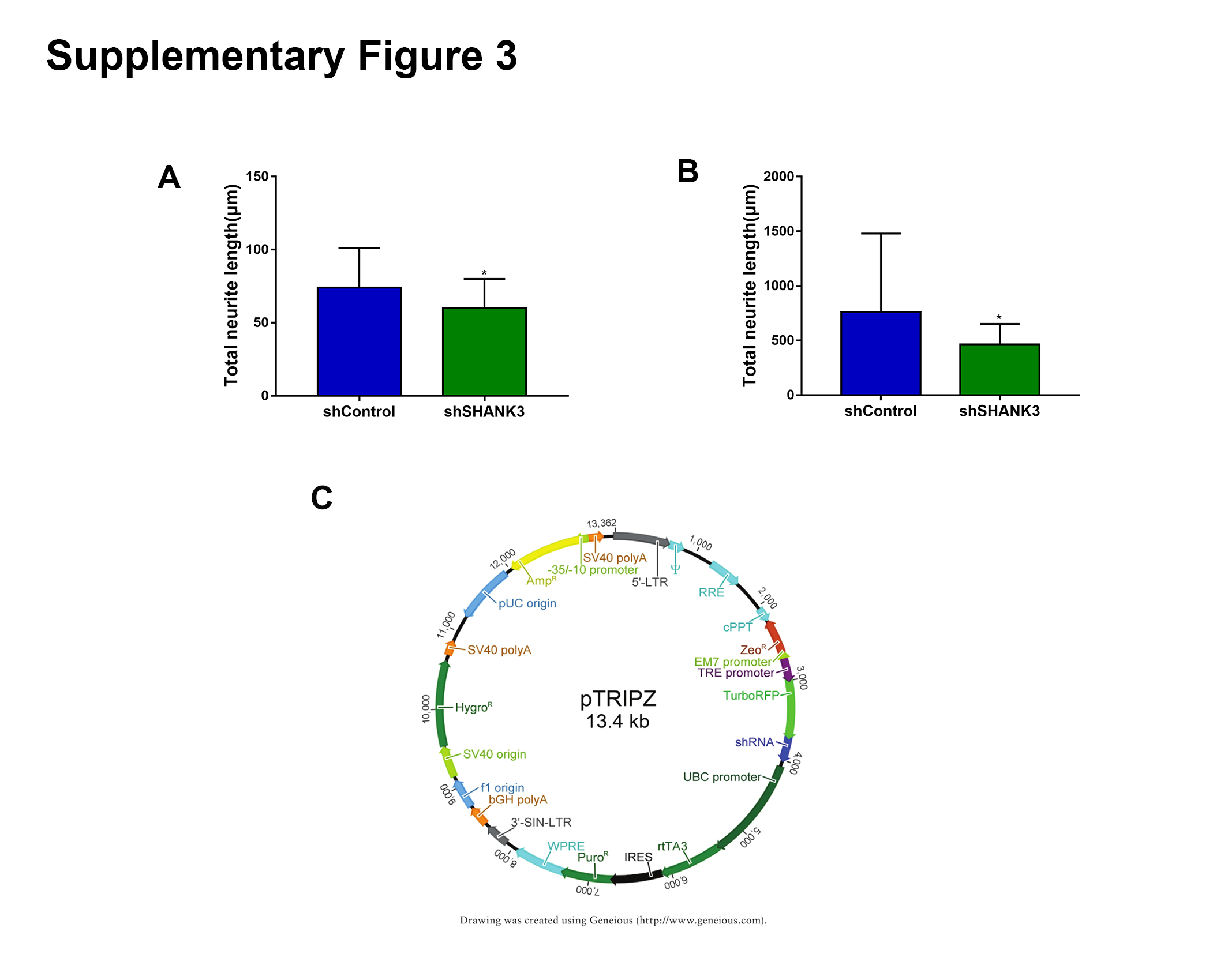

Supplement: FIGURE S3 — Two sets of neural induced model from two separate iPSC lines have been generated in this whole project, and we used one clone of each line to collect data for morphology analysis. Both of these two clone showed significant decrease on dendritic length and soma size after SHANK3 knockdown. Comparison of neurite length in another iPSC line neurons infected with shShank3 and shControl viruses. Morphology reconstructed with ImageJ, statistical significance was evaluated by T-test, data are shown as mean ± SEM. ∗p < 0.05. (A) D9: GABA: n = 35 control and 33 SHANK3 knockdown cells, (p = 0.0037); (B) D9: TH: n = 30 control and 31 SHANK3 knockdown cells, (p = 0.0340); (C) Detailed Vector Map of the pTRIPZ lentiviral vector. [file Image_3.TIF]

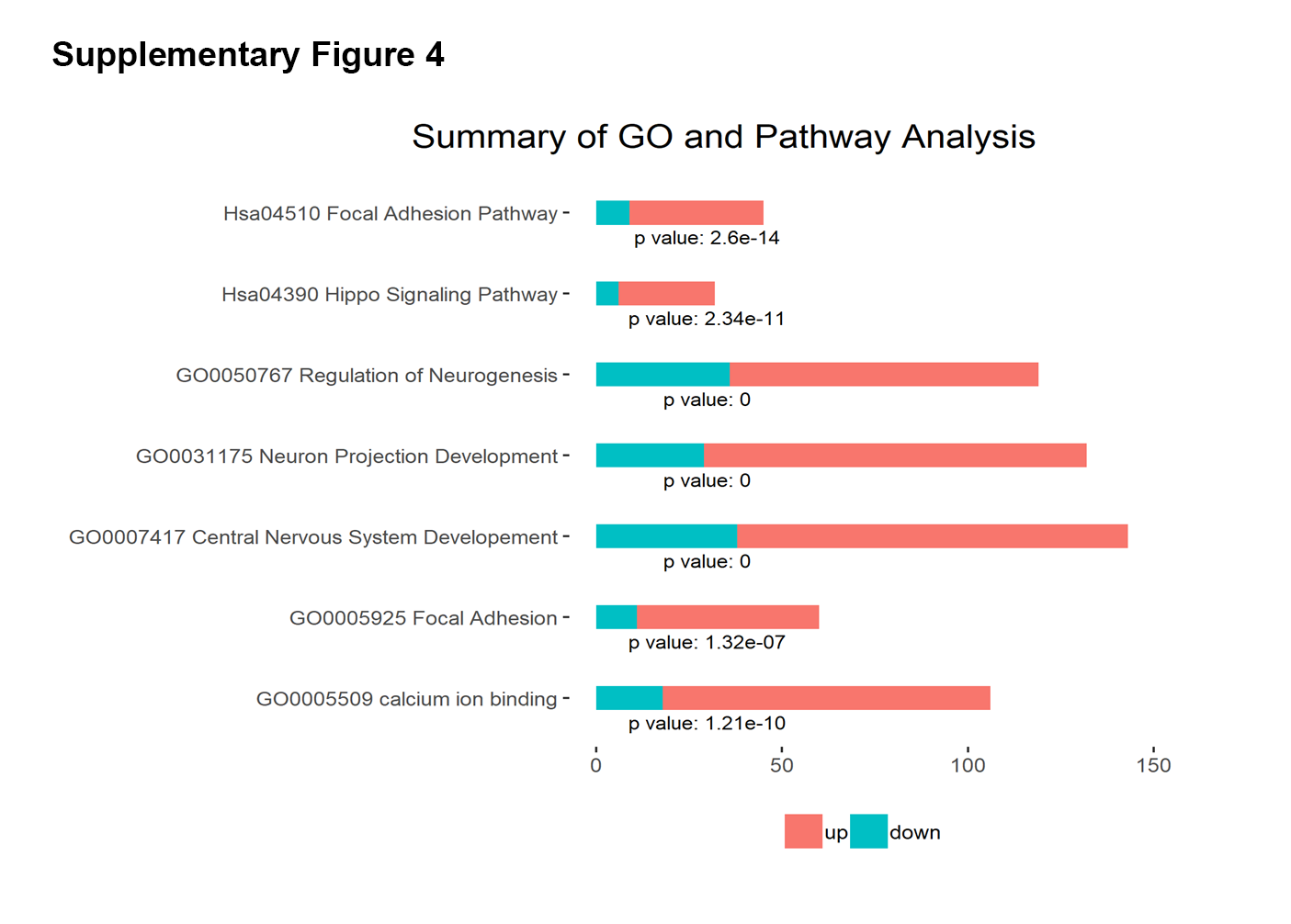

Supplement: FIGURE S4 — Bar charts of gene ontology function categories. [file Image_4.TIF]

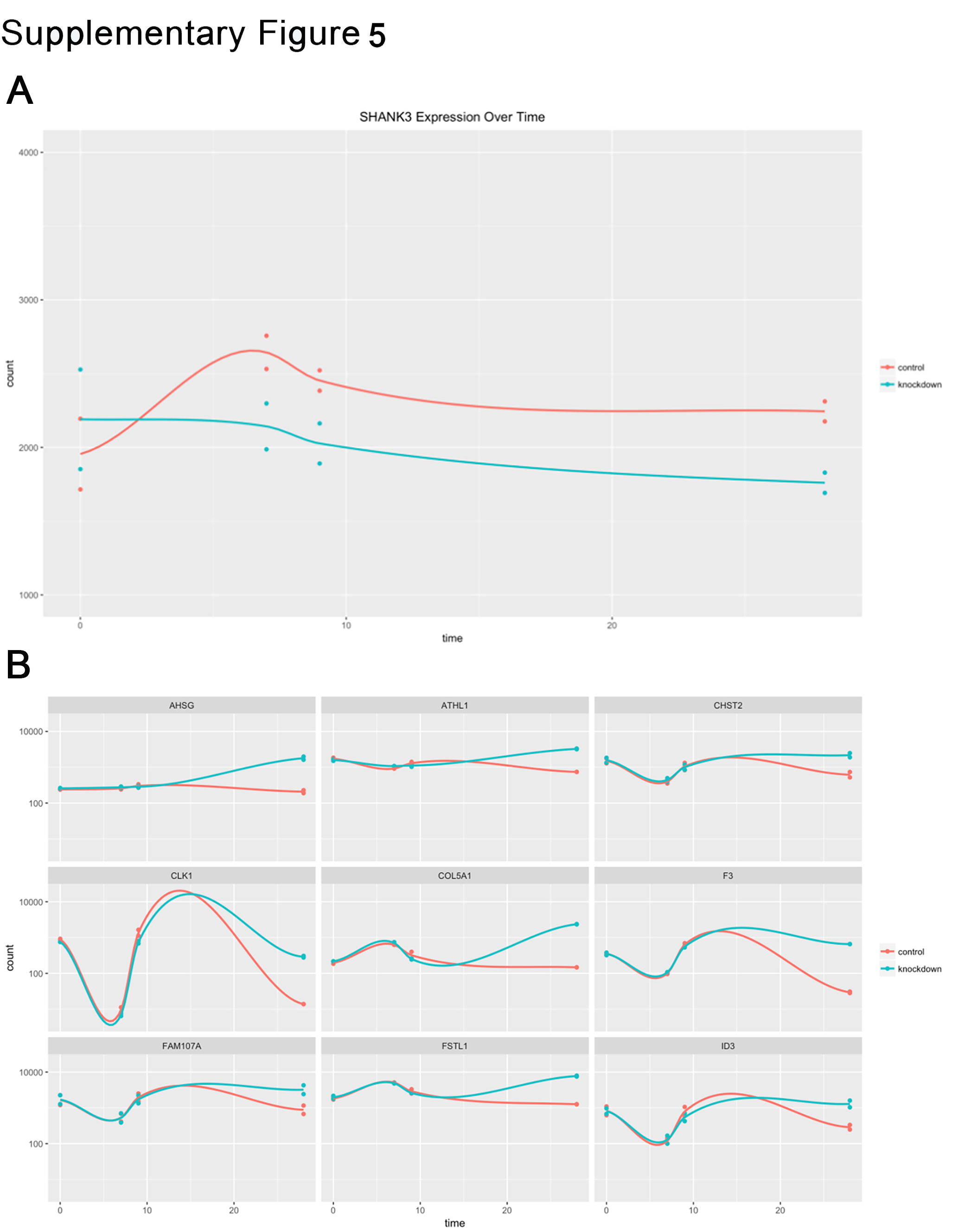

Supplement: FIGURE S5 — Effects of SHANK3 knockdown on gene expression levels. Expression level were transformed into log10 scale. Green lines represent SHANK3 knockdown group and red lines represent control group. (A) Expression level of SHANK3 gene over time between knockdown and control group. (B) Expression level of top 9 genes with smallest p-adj values. [file Image_5.TIF]

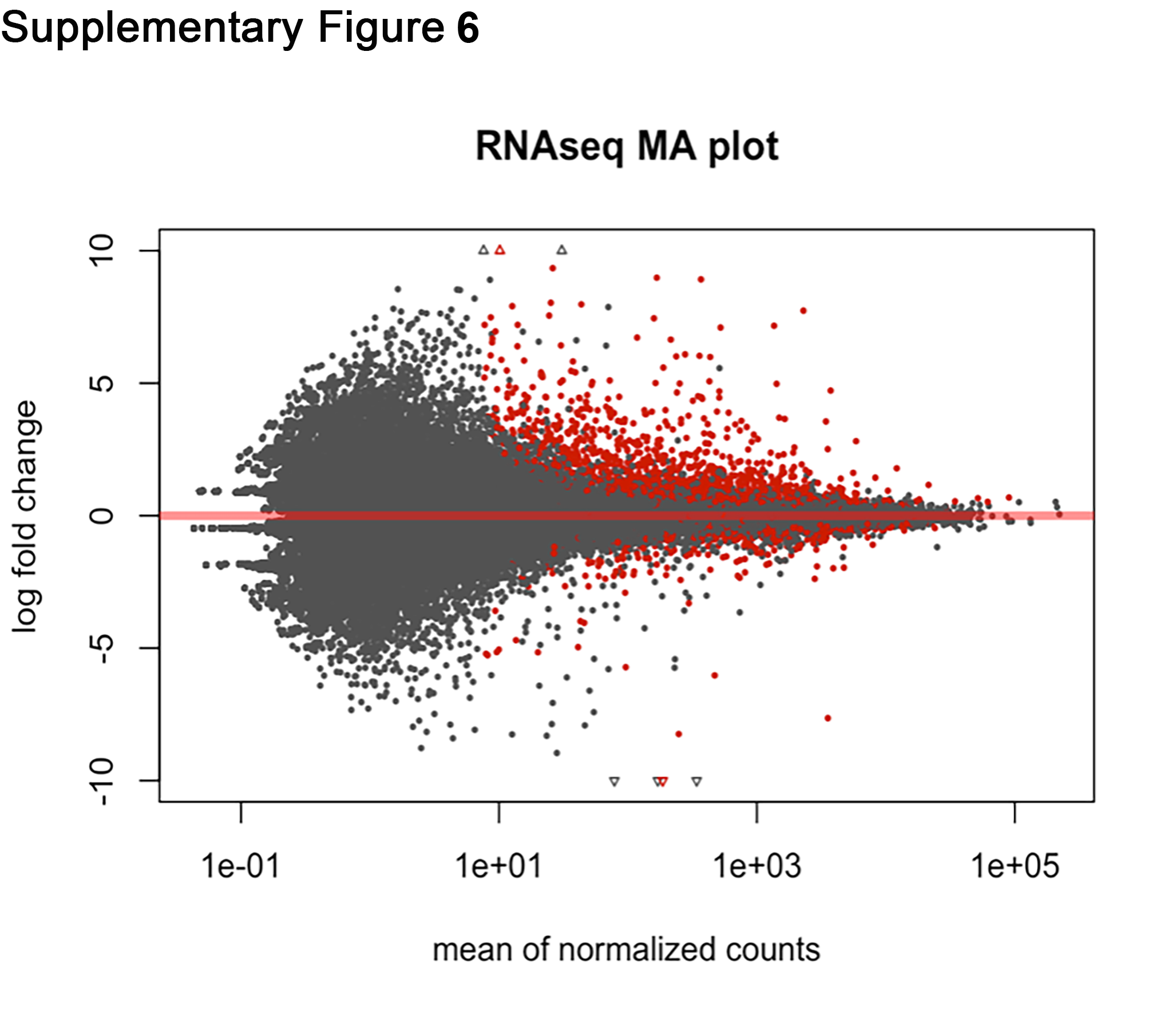

Supplement: FIGURE S6 — RNA sequence data MA plot. Each dot represents a gene. X-axis is the average expression over all samples. Y-axis is the log2 fold change between SHANK3 knockdown and control group. Genes with FDR<0.01 are shown in red. This plot demonstrates that only genes with a large average normalized count contain more information to yield a significant call. [file Image_6.TIF]

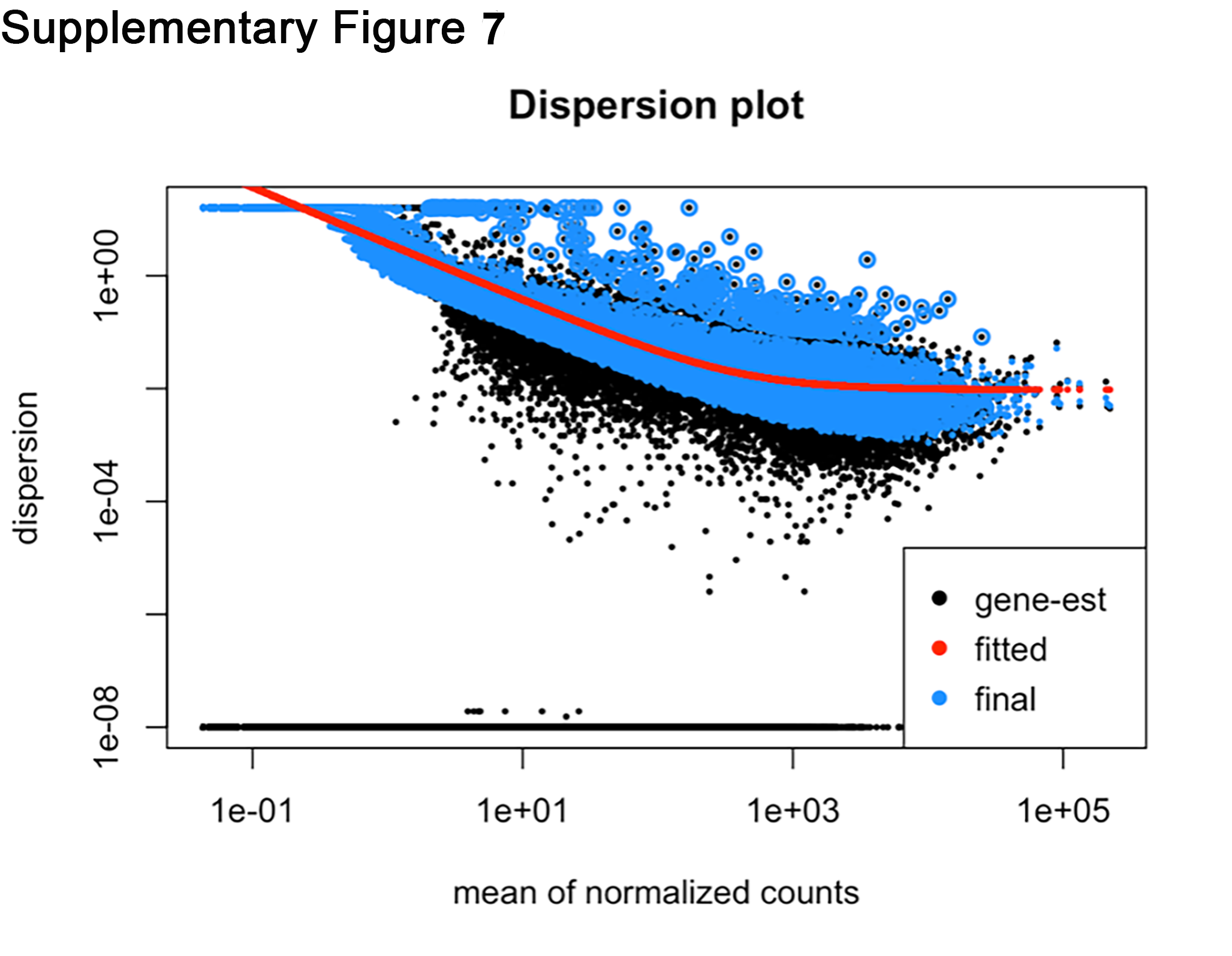

Supplement: FIGURE S7 — Dispersion estimation plot for raw counts data. The dispersion fit is an exponentially decaying curve where dispersion decreased as the counts increased of all genes. Each black point represents the dispersion estimates for each gene across all the eight samples. The red line is fitted trend line, which shows the dispersions’ dependence on the mean. Blue points are the final estimates from black points shrunk to the red fitted line. The blue circles are genes which are labeled as dispersion outliers and are not shrunk toward the fitted trend line. X-axis is the average expression over all eight samples. Y-axis is the dispersion value. The dispersion fit for all genes is an exponentially decaying curve where dispersion decreased with the mean of normalized counts. [file Image_7.TIF]

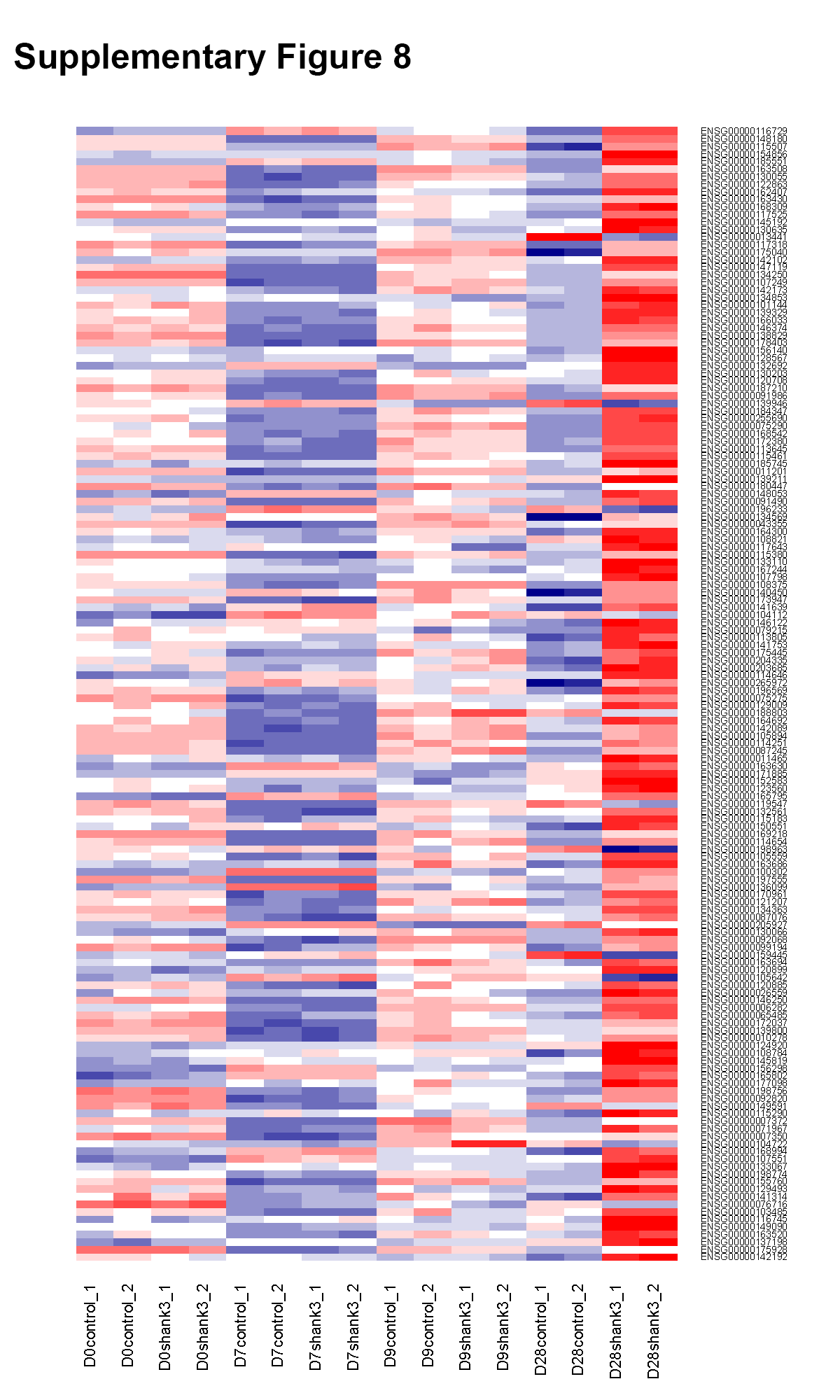

Supplement: FIGURE S8 — The list of top 150 differentially expressed (DE) at each time point between the knockdown and control samples. [file Image_8.TIF]
